# Alteration in the cavity size adjacent to the active site of RB69 DNA polymerase changes its conformational dynamics

**DOI:** 10.1093/nar/gkt674

**Published:** 2013-08-05

**Authors:** Shuangluo Xia, Marcus Wood, Michael J. Bradley, Enrique M. De La Cruz, William H. Konigsberg

**Affiliations:** Department of Molecular Biophysics and Biochemistry, Yale University, New Haven, CT 06520-8114, USA

## Abstract

Internal cavities are a common feature of many proteins, often having profound effects on the dynamics of their interactions with substrate and binding partners. RB69 DNA polymerase (pol) has a hydrophobic cavity right below the nucleotide binding pocket at the tip of highly conserved L415 side chain. Replacement of this residue with Gly or Met in other B family pols resulted in higher mutation rates. When similar substitutions for L415 were introduced into RB69pol, only L415A and L415G had dramatic effects on pre-steady-state kinetic parameters, reducing base selectivity by several hundred fold. On the other hand, the L415M variant behaved like the wild-type. Using a novel tC^o^-tC_nitro_ Förster Resonance Energy Transfer (FRET) assay, we were able to show that the partition of the primer terminus between pol and exonuclease (exo) domains was compromised with the L415A and L415G mutants, but not with the L415M variant. These results could be rationalized by changes in their structures as determined by high resolution X-ray crystallography.

## INTRODUCTION

Replicative DNA polymerases (pols) play a central role in the maintenance of the genetic integrity of all living organisms (1–4). They catalyze the formation of a phosphodiester bond between the 3′-OH group at the primer-terminus and the α-phosphorous atom of an incoming dNTP. The replicative DNA pol from bacteriophage RB69 (RB69pol) is a member of the B family and is responsible for DNA synthesis of both leading and lagging strands of its genome. It has extensive sequence similarity to human replicative pols α and δ and therefore has been regarded as a good model for B family pols (5,6). Although a vast amount of structural and kinetic data have been obtained on RB69pol (7–17), the details of its conformational dynamics, and how it manages to replicate its genome with an error rate less than 10^−^^8^_,_ are still not well understood.

Previously, we have determined high-resolution crystal structures of RB69pol ternary complexes with 8 of the 12 possible mismatches by using a triple mutant (tm, L516A/S565G/Y567A) of RB69pol (9). To capture the missing four purine/purine mismatches, we had to introduce another substitution, L415A, into the triple mutant. We then solved the crystal structures with all 12 mismatches using the quadruple mutant (qm, L415A/L561A/S565G/Y567A). These structures provided new insights into base selectivity and enabled us to propose reasons why incorrect dNTPs are incorporated so inefficiently by wild-type (wt) RB69pol, but the kinetic behavior of this qm was puzzling (15). For example, the maximum turnover rate (k_pol_) for incorporation of a purine dNTP opposite a templating purine base was greater than 300 s^−^^1^, which is faster than the k_pol_ for incorporation of a correct dNTP by wt RB69pol. This is the first example where a mutant replicative DNA pol incorporated an incorrect dNTP much faster than a correct dNTP. This unexpected kinetic behavior was so intriguing that it warranted further investigation. Interestingly, L415 is a highly conserved residue in B family pols. The corresponding Leu to Gly substitution (L604G) in mouse Pol δ caused an increase in genomic instability and further accelerated tumorigenesis (18–20). An equivalent mutation in yeast Pol δ, L612G, produced elevated spontaneous mutation rates (21–23). In addition, replacing L412, the equivalent residue in T4 DNA pol, with Met altered the partition ratio of the primer-terminus between pol and exo domains (24,25). Because of these results, we were curious as to whether similar phenomena would be observed in RB69pol on replacing L415 with Gly, Ala or Met.

**Figure 1.**
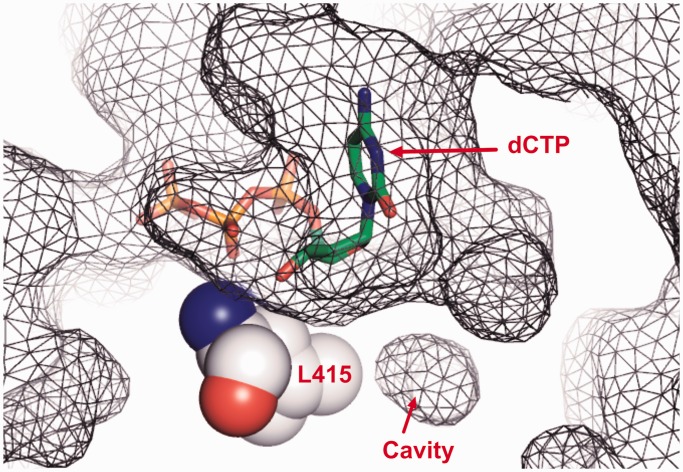
A meshed surface presentation of wt RB69pol ternary complex. The incoming dNTP is shown in green sticks, and L415 is shown in gray space-filling mode. The short red arrow points to the internal cavity.

Our previous reported 1.8 Å resolution structure of wt RB69pol showed that L415 is located right below the triphosphate tail of the incoming dNTP, where a small hydrophobic cavity exists at the tip of the L415 side chain (Figure 1) (17). In many cases, cavities confer flexibility and allow for rapid transitions between structurally distinct states (26). Replicative pols are highly dynamic, as they translocate along a template strand while alternating between open and closed conformations. In addition, these pols remove misincorporated nucleotide residues from the 3′-end of a primer by shuttling it between the pol and exo domains. Recent single molecule studies on several pols suggest that structurally distinct intermediate states exist as well (27,28). Substitution of L415 with residues having smaller side chains, like Ala or Gly, increases the size of this cavity while replacing L415 with Met decreases it. To determine what effects substitutions at this position have on the behavior of RB69pol, we obtained pre-steady-state kinetic parameters for incorporation of correct and incorrect dNMPs by the L415A, L415G and L415M mutants, respectively. In accord with the results obtained with pol δ, the L415A and L415G RB69pol mutants incorporated incorrect dNMPs with an efficiency that was more than a 100-fold greater than wt RB69pol. In contrast, the catalytic efficiency for incorporation of incorrect dNMPs by the L415M mutant was comparable with that of wt RB69pol. We also determined structures of the ternary complex of L415A, L415G and L415M mutants, respectively, at resolutions ranging from 1.80 to 2.04 Å. The structural features observed with these three mutants are consistent with, and can be used to rationalize, their kinetic behaviour. In addition, we developed a tC^o^-tC_nitro_ FRET assay to determine how the primer-terminus shuttles between the pol and exo domains. We found that L415A and L415G mutants were not able to differentiate fully complementary primer/templates (P/Ts) from those with a mispair at the P/T terminus.

## MATERIALS AND METHODS

### Materials

**Figure 2.**
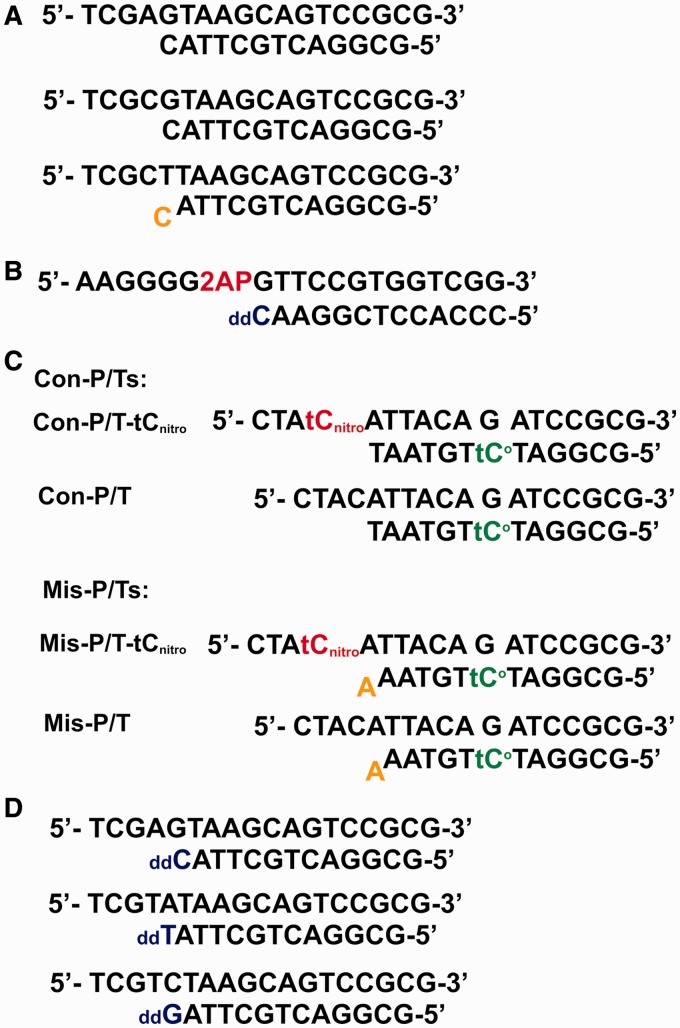
P/T sequences used in this study. (**A**) P/T sequences used in chemical quench experiments. (**B**) P/T sequence used in the fluorescence titration assay. 2AP is 2-amino purine, and _dd_C is dideoxy-dC. (**C**) P/T sequences used in tC^o^-tC_nitro_ FRET assay. (**D**) P/T sequences used for X-ray crystallography. _dd_T and _dd_G are dideoxy-dT and dideoxy-dG, respectively.

All oligonucleotides were synthesized at the Keck facilities (Yale University) and purified via polyacrylamide gel electrophoresis. The 2-amino purine (2AP), tC^o^ and tC_nitro_ phosphoramidites were from Glen Research. The sequences of the primer-templates (P/Ts) used in this study are shown in Figure 2. *Escherichia coli* strain BL21 GOLD (DE3) and DH5α were from Invitrogen. The dNTP stock solutions (100 mM) and EDTA-free protease inhibitor cocktail tablets were from Roche. [γ-^32^P]-ATP was from Perkin Elmer Life Science. Ni-NTA resin was from Qiagen, and the Q-Sepharose column was from GE Healthcare. T4 polynucleotide kinase was from New England Biolabs. Other chemicals were analytical grade from Sigma Aldrich.

### Constructing and purifying RB69pol L415 variants

RB69pol L415 variants (L415A, L415G and L415M) in an exonuclease-deficient background (D222A and D327A) were constructed by using the QuickChange site-directed mutagenesis method (Stratagene). Plasmids containing the desired mutations, as confirmed by sequencing, were transformed into BL21 GOLD (DE3) cells. Overexpression of the mutant pol was induced when the culture OD_600_ reached 0.6 by addition of IPTG to 0.5 mM, and the desired protein was expressed overnight at 17°C. After low-speed centrifugation of the culture, the resulting cell pellets were frozen in liquid nitrogen. Frozen cell pellets were suspended in a lysis buffer [20 mM Tris–HCl (pH 7.4), 200 mM NaCl, 2 mM β-mercaptoethanol, protease inhibitor mixture] and passed through a microfluidizer followed by centrifugation at 40 000 rpm for 30 min. The supernatant was applied to a Ni-NTA column and eluted with a linear gradient of increasing [imidazole]. Peak fractions were pooled and dialyzed against Q buffer A [20 mM Tris–HCl (pH 7.4), 20 mM KCl, 5% (v/v) glycerol, 2 mM DTT]. The samples were then loaded onto a Source Q column and eluted with a 20–500 mM KCl linear gradient. The purest pol-containing fractions, as determined by SDS–PAGE, were pooled and dialyzed against 20 mM Tris–HCl (pH 7.4), 100 mM NaCl, 5% (v/v) glycerol and 2 mM β-mercaptoethanol. The protein was concentrated to 10 mg/ml and stored at −80°C until use.

### Rapid chemical quench experiments

All rapid chemical quench experiments were performed at 23°C using a KinTek RFQ-3 instrument (KinTek Corp., University Park, PA). The reaction mixture contained 66 mM Tris–HCl (pH 7.4) and 10 mM MgSO_4_. Single-turnover conditions were used with a 10-fold excess of pol over the P/T (sequences of P/T are shown in Figure 2A). Briefly, pol and P/T complex from one syringe were rapidly mixed with various [dNTP] from the other syringe for various times. The concentrations of pol and P/T after mixing were 1 µM and 83 nM, respectively. Reactions were quenched with 0.5 M EDTA (pH 8.0). Products were separated by 19:1% (w/v) PAGE containing 8 M urea, visualized with a MD Storm 860 imager (Molecular Imaging) and quantified using ImageQuaNT software. For each K_d,app_ and k_pol_ determination, six different [dNTP] were used. Data from single turnover experiments were fit to a single exponential equation: Y = A × [1 – exp(-k_obs_ × t)], where Y is the concentration of DNA product, k_obs_ is the observed rate constant. The [dNTP] dependence of k_obs_ was fitted to a hyperbolic equation: k_obs_ = k_pol_ × [dNTP]/[K_d,app_ + [dNTP]], where k_pol_ is the maximum turnover rate constant and K_d,app_ is the apparent dissociation constant. The corresponding standard deviations shown in Table 2 were calculated from data fitting using Grafit5.0 (Erithacus Inc.).

### Equilibrium fluorescence binding titrations

Fluorescence emission spectra of 250 nM dideoxy-terminated P/T (2AP at the n position of the template strand, Figure 2B) with wt RB69pol or L415 variants (1 μM), 50 mM Tris–HCl (pH 7.4), 2 mM MgSO_4_, and varying [dNTP] were recorded at 25°C using a Photon Technology International scanning spectrofluorometer. We acquired the spectra by exciting the sample at 315 nm and collecting emissions from 330 to 445 nm. Peak intensities at 365 nm were plotted against the [dNTP], then fit to a hyperbolic equation: F = A [dNTP]/(K_d,net_ + [dNTP]) + C, where F is the observed fluorescence intensity, A is the maximum change in fluorescence intensity, C is the fluorescence in the absence of dTTP and K_d,net_ is the net dissociation constant for dTTP binding; or a quadratic equation: F = F_max_ × [1- (E_o_ + S_o_ + K_d,net_ – sqrt((E_o_ + S_o_ + K_d,net_)^2^ – 4 × E_o_ × S_o_)/(2 × E_o_)] + C, where F_max_ is the maximum value for the fluorescence change at saturating [dTTP], E_o_ is the total [pol] and S_o_ is the total [dNTP]. The intensities were corrected for the intrinsic fluorescence of the buffer and pol solutions.

### Stopped-flow fluorescence experiments

Transient kinetic fluorescence experiments were carried out using an Applied Photophysics (Leatherhead, UK) SX18MV-R stopped-flow apparatus thermostatted at 24°C. The excitation wavelength for 2AP is 314 nm. A 345 nm long pass glass filter was used for monitoring dAP fluorescence emission. A solution containing 1 μM pol, 250 nM dideoxy-terminated P/T (Figure 2B), 50 mM Tris–HCl (pH 7.4), 2 mM MgSO_4_ mixed rapidly with varying concentration of dTTP. The fluorescence intensities were corrected for the intrinsic fluorescence of the pol and buffer solutions. All time courses were the average of five runs at each [dNTP]. Then each time course was fit to equation: F = Σ[A_i_ × exp(-k_obs,i_ × t)] + C, (i = 1 to n), where F is the observed fluorescence intensity, A is the maximum change in fluorescence intensity, k_obs_ is the observed rate constant and C is the offset constant. The change of amplitude was plotted against [dNTP], then fit to a hyperbolic or quadratic equations as shown in the ‘Materials and Methods’ section of equilibrium fluorescence titration.

### The tC^o^-tC_nitro_ FRET assay

Fluorescence emission spectra of 200 nM P/T (Figure 2D) with wt RB69pol or the L415 variants (2 μM) in a solution containing 10 mM Tris–HCl (pH 7.4), 50 mM NaCl, 2 mM MgSO_4_, were recorded at 25°C using a Photon Technology International scanning spectrofluorometer. We acquired the spectra by exciting the sample at 375 nm and collecting emissions from 395 to 645 nm. Peak intensities at 450 nm were used in the FRET calculation. The FRET efficiency (E) was represented by a donor quenching efficiency, defined by the equation: E = 1− I_DA_/I_D_, where I_DA_ is the fluorescence intensity of the donor (tC^o^) in the presence of the acceptor (tC_nitro_) and I_D_ is the fluorescence intensity in the absence of acceptor. The *P*-value was calculated from three independent measurements.

### Crystallization of ternary complexes of RB69pol L415 variants

Each RB69pol L415 variant was mixed in an equimolar ratio with dideoxy terminated P/T (Figure 2D) to give a final protein concentration of 110 µM. The incoming dATP or dTTP was then added to give a final concentration ∼2 mM. Crystals of the ternary complex were grown under Al’s oil by mixing a solution containing 100 mM CaCl_2_, 15% (w/v) PEG 350 monomethyl ether and 100 mM sodium cacodylate (pH 6.5) with an equal volume of the protein complex. The square rod-shaped crystals grew in 3 days at 20°C to a size of ∼100 × 120 × 120 µm. Crystals were transferred from the mother liquor to a cryoprotectant solution with a high concentration of PEG 350 monomethyl ether (30% w/v) before freezing in liquid nitrogen.

### Data collection, structure determination and refinement

All X-ray diffraction data were collected using the synchrotron radiation sources at beam line 24ID-E, Northeast Collaborative Access Team, Advanced Photon Source, Argonne National Laboratory (APS, ANL, Chicago, IL). The data were processed using HKL2000 program suite (Table 1) (29). The structures were determined by molecular replacement using Phaser (30), starting with our previously reported wt RB69pol structure of the ternary complex (3NCI) and refined using REFMAC5 (31). The resolution cutoff for L415M mutant is limited by the data collection setup. The P/T duplex and the incoming dNTP were subsequently built using the program COOT (32). Structure refinement statistics are summarized in Table 1. All figures presented were made using the program Pymol (33).

**Table 1.** Crystallographic statistics for data collection and structure refinement of ternary complexes of RB69pol L415 variants

Parameters	L415A	L415G	L415M
Space group	P2_1_2_1_2_1_	P2_1_2_1_2_1_	P2_1_2_1_2_1_
Unit cell [^a^^,^^b^^,^^c^(Å)]	75.0,120.3,130.6	75.6,120.5,130.9	75.0,120.5,130.8
Resolution (Å)	50.0–1.80	50.0–2.04	50.0–2.02
No. of unique reflections	108 938	71 014	76 223
Redundancy	3.2 (3.2)	3.0 (2.9)	3.2 (3.0)
Completeness (%)	99.1 (98.9)	93.6 (93.8)	97.1 (91.7)
R_merge_ (%)	7.1 (76.2)	8.1 (74.1)	5.8 (36.6)
I/σ	16.2 (1.2)	12.9 (1.5)	16.6 (2.8)
Final model			
Amino acid residues	903	903	903
Water molecules	854	722	502
Ca^2+^ ions	6	6	6
Template nucleotides	18	18	18
Primer nucleotides	13	13	13
dNTP	1	1	1
Refinement statistics			
Reflections	103 190	57 504	72 335
R (%)	17.8 (28.2)	17.1 (25.0)	17.9 (21.1)
R_free_ (%)	21.1 (31.3)	21.9 (28.6)	21.3 (24.4)
r.m.s.d			
Bond length (Å)	0.007	0.006	0.006
Bond angles (°)	1.104	1.054	1.059
PDB code	4J2A	4J2B	4J2E

^a^Statistics for the highest resolution shell are in parenthesis.

^b^*R*_merge_ = Σ*_hkl_* Σ_j_|I_j_(*hkl*)−<I(*hkl*)>|/Σ*_hkl_*<I(*hkl*)>, statistics for merging all observations for given reflections.

^c^R = Σ*_hkl_* |F_obs_(*hkl*)-F_calc_(*hkl*)|/Σ*_hkl_*ΣF_obs_(*hkl*), statistics for crystallographic agreement between the measured and model-calculated amplitudes. R_free_ is the agreement for cross-validation data set.

^e^Root mean squares deviations (rmsd) to ideal values.

### Data deposition

Atomic coordinates and structure factors have been deposited in the PDB bank with accession codes 4J2A (L415A ternary complex), 4J2B (L415G ternary complex) and 4J2E (L415M ternary complex).

## RESULTS AND DISCUSSION

### Pre-steady-state kinetic parameters for incorporation of dNMP into DNA and primer extension beyond a mispair by the L415 variants of RB69pol

As L415 is a highly conserved residue in B family pols and because its replacement caused profound changes in the behavior of other pols, we decided to see how L415 substitutions would affect RB69pol. Accordingly, we replaced L415 by Gly, Ala or Met. These were the residues that were substituted for L612 in human pol delta and L412 in T4pol (22,25). Although the side chain of L415 does not directly interact with the incoming dNTP or with the P/T duplex, we wanted to determine how RB69pol would process correct and incorrect incoming dNTPs when L415 was replaced with either residues having smaller side chains or with a residue bearing a longer side chain than Leu. Accordingly, we determined the pre-steady-state kinetic parameters for incorporating dTMP opposite dA with the L415G, L415A and L415M mutants. As shown in Table 2, the maximum turnover rates (k_pol_) for incorporating a correct dNMP by the L415A and L415G mutants were 11 s^−^^1^ and 58 s^−^^1^ respectively, which were 5- to 25-fold lower than the values found with wt RB69pol. The apparent dissociation constants (K_d,app_) were 2.7 and 9 µM, respectively, for L415A and L415G mutants, which were 5- to 15-fold lower than the values observed with wt RB69pol. Consequently, the corresponding catalytic efficiencies (k_pol_/K_d,app_) with L415A and L415G mutants are comparable with wt RB69pol. In contrast, the maximum turnover rate increased by 30%, and the apparent dissociation constant decreased by 70% when Leu415 was replaced by Met. As a result, the L415M mutant incorporated dTMP opposite dA 5-fold more efficiently than wt RB69pol.

**Table 2.** Pre-steady-state kinetic parameters for incorporation of correct and incorrect dNMPs by wt RB69pol and the L415 variants

Pols	k_pol_ (s^-1^)	K_d,app_ (µM)	k_pol_/K_d,app_ (µM^−1^ s^−1^)	Discrimination
Correct BP	dTTP/dA			
wt	270	42	6.4	
L415A	11 ± 1	2.7 ± 0.6	4.0	
L415G	58 ± 1	9 ± 0.6	6.4	
L415M	375 ± 10	12 ± 1	31.2	

Pu-Pu BP	dATP/dA			
wt	0.13	810	1.6 × 10^−4^	4.0 × 10^4^
L415A	15 ± 1	640 ± 150	2.3 × 10^−2^	1.7 × 10^2^
L415G	1 ± 0.01	220 ± 20	4.5 × 10^−3^	1.4 × 10^3^
L415M	0.03 ± 0.003	1300 ± 270	2.6 × 10^−5^	1.2 × 10^6^

Py-Pu BP	dCTP/dA			
wt	N.A.D	>2000	1.7 × 10^−4^	3.7 × 10^4^
L415A	N.A.D	>2000	6.0 × 10^−3^	6.7 × 10^2^
L415G	1.6 ± 0.1	1330 ± 200	1.2 × 10 ^− 3^	5.3 × 10^3^
L415M	N.A.D	>2000	5.4 × 10^−5^	5.7 × 10^5^

Py-Py BP	dTTP/dC			
wt	0.09	1700	5.2 × 10^−5^	1.2 × 10^5^
L415A	130 ± 1	1700 ± 20	7.6 × 10^−2^	5.3 × 10^1^
L415G	N.A.D	>2000	1.1 × 10^−2^	5.8 × 10^2^
L415M	N.A.D	>2000	1.4 × 10^−4^	2.2 × 10^5^

Pu stands for purine; Py stands for pyrimidine; BP stands for base-pair; N.A.D stands for not accurately determined because the corresponding K_d,app_ value is greater than 2 mM, and therefore only the k_pol_/K_d,app_ values are reliable. Discrimination is defined by (k_pol_/K_d,app_)_correct_/(k_pol_/K_d,app_)_incorrect_. Representative gel, progress curve, and plot of k_obs_ versus (dTTP)] are shown in Supplementary Figure S1.

Our previous studies with mismatched-containing complexes showed that the 12 combinations of mismatched base pairs can be categorized into three groups based on hydrogen-bonding patterns and geometric shapes: (i) purine-purine; (ii) purine-pyrimidine; and (iii) pyrimidine-pyrimidine (15). Therefore, we chose one mismatched base pair from each group to represent incorrect nucleotide incorporation. We then determined the pre-steady-state kinetic parameters for incorporating dAMP opposite dA, dCMP opposite dA and dTMP opposite dC by all three L415 mutants. The results in Table 2 show that replacement of Leu415 with Gly caused the catalytic efficiencies for incorporation of a purine opposite a purine (dAMP/dA), a pyrimidine opposite a purine (dCMP/dA) and a pyrimidine opposite a pyrimidine (dTMP/dC) to increase by 28-, 7- and 210-fold, respectively. The corresponding base selectivity decreased by 7- to 200-fold relative to wt RB69pol. Similar patterns were observed for the L415A variant. The major difference between the two mutants was that the L415A mutant incorporated incorrect dNMPs 5- to 7-fold more efficiently than the L415G mutant. In particular, the k_pol_ for incorporation of dTMP opposite dC was 130 s^−^^1^, which is three orders of magnitude greater than the value observed with wt RB69pol. Overall, the base selectivity of L415G decreased by 50- to 2200-fold compared with wt RB69pol. Interestingly, when L415 was replaced with Met, an amino acid with a longer side-chain, the base selectivity was not compromised. In contrast, the discrimination improved by 10- and 100-fold for the incorporation of dCMP and dAMP opposite dA, respectively. It would appear that the L415M mutant is more selective than wt RB69pol. This was surprising, as the equivalent substitution, L412M, in T4 DNA pol has been reported to exhibit an elevated mutation rate.

For extension past a mismatch, we determined the kinetic parameters for incorporation of dTMP opposite dA when there was a T/C pair at the P/T junction. As shown in Table 3, both L415A and L415G mutants bypassed the mismatch more efficiently than the wt pol. The corresponding catalytic efficiencies for extension past T/C pair are 95- and 43-fold higher than the value observed with wt RB69pol. In contrast, the efficiency for extension past the mismatch by the L415M mutant is only slightly higher (5-fold) than that of wt RB69pol. Overall, replacing L415 with Ala or Gly resulted in mutant pols that are more error-prone.

**Table 3.** Catalytic efficiency for extension beyond a mispair by wt RB69pol and L415 variants

Parameters	wt	L415A	L415G	L415M
k_pol_ (s^−1^)	N.A.D	19 ± 1	46 ± 4	N.A.D
K_d_ (µM)	>2000	245 ± 30	1300 ± 270	>2000
k_pol_/K_d_ (µM^−1^ s^−1^)	8.1 × 10^−4^	7.7 × 10^−2^	3.5 × 10^−2^	4 × 10^−3^

N.A.D stands for not accurately determined because the corresponding K_d,app_ value is greater than 2 mM, and therefore only the k_pol_/K_d,app_ value is reliable.

### Net binding affinity of dTTP by the L415 variants of RB69pol

**Figure 3.**
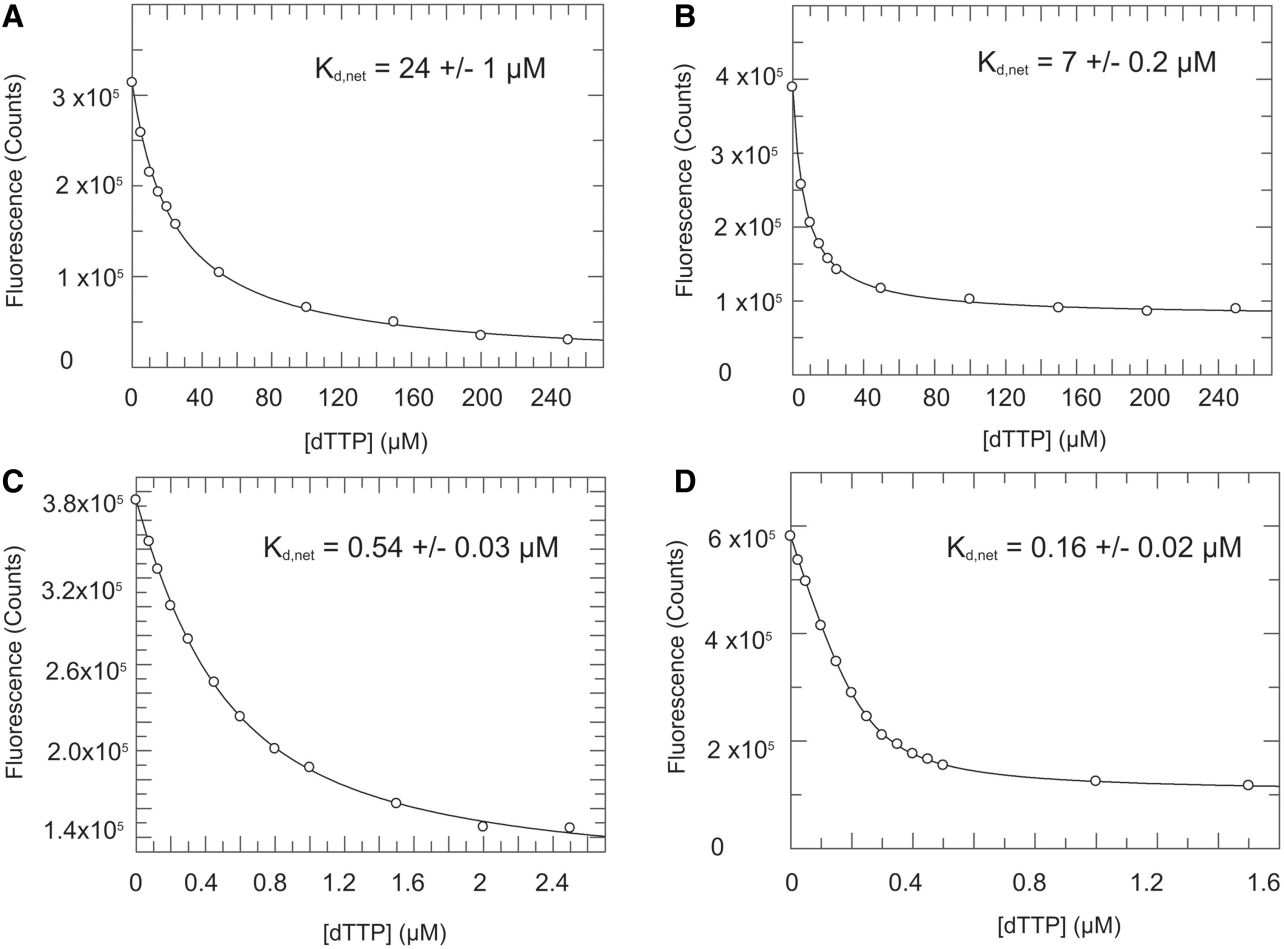
Equilibrium fluorescence quenching titrations of dTTP opposite 2AP in P/T complexes with (**A**) wt RB69pol; (**B**) L415M; (**C**) L415G; and (**D**) L415A variant.

**Scheme 1.**
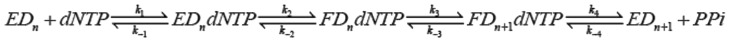
Minimal kinetic scheme for dNTP binding and incorporation. ED_n_ is the open complex, and FD_n_ is the closed complex.

The apparent dissociation constant (K_d,app_) determined from rapid quench experiments does not truly reflect the net binding affinity of an incoming dNTP. Our previous structural and kinetic studies with 2AP, a fluorescent adenine analog, have shown that 2AP fluorescence is quenched as dTTP binds to an RB69pol-P/T binary complex with 2AP as the templating base and a dideoxy-terminated ddC at the 3′-end of the primer (16,34). We have interpreted the fluorescence quenching by dTTP as a consequence of 2AP stacking with the penultimate base pair as the fingers domain closes. The dTTP concentration-dependent equilibrium fluorescence quenching curves (Figure 3) were fit to a hyperbolic or quadratic equation to estimate the net equilibrium dissociation constant (K_d,net_). The K_d,net_ is defined as K_d,1_/(1 + K_2_), where K_d,1_ is the ground-state dissociation constant and K_2_ is the equilibrium constant for the isomerization step according to Scheme 1. As shown in Figure 3, the K_d,net_ of dTTP binding to the L415A, L415G and L415M mutants are 0.16, 0.54 and 7 μM respectively, which are 95-, 40- and 3-fold lower than the K_d,net_ observed with wt RB69pol. Thus, replacing L415 with Gly or Ala significantly enhances the binding affinity of incoming dNTPs to the RB69pol-P/T binary complex.

As an independent check of these results, transient kinetic experiments were performed using stopped-flow fluorescence. With 2AP at the templating position, the RB69pol-P/T binary complex was rapidly mixed with increasing concentrations of dTTP. The fluorescence amplitude changes are plotted against dTTP concentration. As shown in Supplementary Figure S2, the best-fit K_d,net_ values are 0.15, 0.42 and 13 μM for L415A, L415G and L415M, respectively. These values are close to the K_d,net_ values determined from equilibrium 2AP fluorescence titrations. Interestingly, when the concentration of dTTP is 100-fold greater than the concentration of the RB69pol-P/T binary complex, full quenching of 2AP fluorescence occurred within the dead time of the instrument (<2 ms) for wt RB69pol and for all three L415 mutants (Supplementary Figure S3B). In other words, the rate of 2AP fluorescence quenching corresponding to the initial binding and fingers closing steps for wt and the three mutant pols were greater than 500 s^−^^1^. According to the results from the pre-steady-state kinetic experiments, the maximum turn-over rate for the L415A and L415G mutants were only 11 s^−^^1^ and 58 s^−^^1^, respectively. We define the chemistry step as formation of the covalent bond, which happens instantaneously. These results strongly support the existence of a rate-limiting step after fingers closing, but before chemistry, which is likely to be the rearrangement of active-site residues.

### Using tC^o^-tC_nitro_ FRET pair to monitor partition of the primer terminus between the pol and exo domains

**Figure 4.**
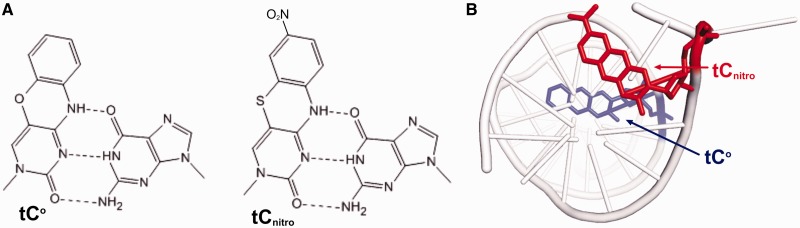
Structures of tC^o^ and tC_nitro_. (**A**) Pairing of guanine and tC^o^. (**B**) Pairing of guanine and tC_nitro_. (**C**) The modelled tC^o^ and tC_nitro_ FRET pair in a P/T duplex. tC^o^ is shown in red, and tC_nitro_ is shown in blue.

It has been reported that the L412M mutant of T4 pol is defective in switching between pol and exo domains (24). As RB69pol is closely related to T4 pol, we wanted to determine whether pol-exo partitioning was compromised in the RB69pol L415 variants. Wilhelmsson’s group has developed an all-nucleobase FRET pair consisting of tC^o^ as the donor and tC_nitro_ as the acceptor, which we have adopted for this purpose (35,36). As shown in Figure 4A, both tC^o^ and tC_nitro_ are cytosine analogues and can form three HBs with guanine. One distinctive feature of this FRET pair is its well-defined orientation when embedded in a DNA duplex (35,36). It provides an excellent control for the orientation factor in FRET efficiency calculations. Accordingly, we designed two sets of duplex DNAs (Figure 2C). One set acted as the control P/T (Con-P/T), which was a matched duplex. The other set had an A/A mismatch at the n-1 position (Mis-P/T). The donor, tC^o^, was located at the n-7 position in the primer strand, and the acceptor, tC_nitro_, was placed at the N position of the template strand. The relative orientation of tC^o^-tC_nitro,_ when embedded in the P/T is shown in Figure 4B and is modelled on our previously reported dGTP/tC^o^-containing structure of RB69pol (10). The resulting FRET efficiencies are shown in Table 4. For an isolated DNA duplex, FRET efficiency decreases as the duplex at P/T junction unwinds due to the presence of mismatches. The FRET efficiency for the control P/T is 0.43. The value drops to 0.32 for Mis-P/Ts. Interestingly, on binding the wt RB69pol, the FRET efficiency for both P/Ts decrease. The changes are more dramatic for P/Ts with mismatches. The corresponding FRET efficiencies for the control P/T and the Mis-P/T are 0.37 and 0.18, respectively, indicating that RB69pol responds to local conformational changes at the P/T junction. The FRET efficiency for Mis-P/T with wt RB69pol is 0.18, which is 44% lower than that of the Mis-P/T alone, suggesting that RB69pol switched the primer end of Mis-P/T from the pol to the exo domain; hence, the separation between tC^o^ and tC_nitro_ FRET pair increases. This separation does not occur unless the P/T is in a binary complex with the pol. This observation confirms that tC^o^ and tC_nitro_ FRET pair method is sensitive enough to detect the local conformational changes at the P/T junction and can be used to monitor partitioning of DNA substrates between the pol and exo domains.

**Table 4.** FRET efficiency for tC^o^-tC_nitro_ pair in binary complexes with wt RB69pol and L415 variants

P/T	P/T alone	wt-P/T	L415A-P/T	L415G-P/T	L415M-P/T
Con-P/T	0.43 ± 0.01	0.37 ± 0.01	0.20 ± 0.02	0.19 ± 0.02	0.35 ± 0.05
Mis-P/T	0.32 ± 0.04	0.18 ± 0.04	0.23 ± 0.02	0.21 ± 0.02	0.24 ± 0.05
*P*-value	0.01	0.002	0.07	0.18	0.04

Representative fluorescence spectra of various tC^o^-containing P/Ts in the absence and presence of pol are shown in Supplementary Figure S4.

We then determined the FRET efficiency for the control P/T and Mis-P/Ts in the presence of each of the three L415 variants. Upon substitution of L415 with Gly, the FRET values were 0.19 and 0.21 for control P/T and Mis-P/T respectively. The difference between these two averaged FRET efficiency values is not statistically significant from the three independent measurements (*P*-value = 0.18). Therefore, the FRET values for both P/Ts in the presence of the L415G mutant are not distinguishable so it appears that the L415G mutant cannot distinguish the control P/T from the Mis-P/T. Interestingly, the L415A mutant follows the same pattern. The FRET values for control P/T and Mis-P/T were 0.20 and 0.23, respectively, with a corresponding *P*-value of 0.07. This is consistent with our kinetic data in that the L415A and L415G mutants are almost two orders of magnitude more efficient at incorporating a nucleotide residue beyond a mispair. In contrast, the corresponding FRET values for the control P/T and the Mis-P/T in the presence of L415M mutant were 0.35 and 0.24, respectively. Although the FRET value 0.24 for the Mis-P/T was 0.06 units higher than the value observed with wt RB69pol, it is still significantly below the FRET value 0.35 for the control P/T (the corresponding *P*-value was 0.04), suggesting that the L415M mutant is still sensitive to the local conformational changes at the P/T junction. Therefore, the partitioning between the pol and exo domains is not significantly compromised with the L415M mutant. This observation is consistent with our pre-steady-state kinetic data as; (i) the L415M mutant bypasses mismatches slightly better than wt RB69pol and; (ii) base selectivity of the L415M mutant is comparable with that of wt RB69pol.

### Structural overviews of the ternary complexes of L415 variants

To provide a structural basis that could account for the different kinetic behavior observed among L415 variants and wt RB69pol, we determined the crystal structures of three ternary complexes: dTTP/dA-with the L415A mutant; dATP/dT-with the L415G mutant and dATP/dT-with the L415M mutant with resolutions ranging from 1.80 to 2.04 Å and R_free_ values ranging from 21.3% to 21.9% (Table 1). The overall structures of these three ternary complexes are close to that of our reported 1.8 Å dCTP/dG-containing wt RB69pol ternary complex, with root-mean-square deviations of Cα atoms varying from 0.21 to 0.26 Å. The electron densities for the incoming dNTP, the P/T duplex and the surrounding network of ordered water molecules are well defined (Supplementary Figure S5A–C). In particular, the five ordered water molecules that have been previously reported to recognize the O2 of pyrimidine or N3 of purine as hydrogen bond acceptors in the minor groove of the P/T duplex have been consistently observed in all three structures. All of these water molecules serve as extensions of amino acid side chains, such as Y416, Y567 and T622, and mediate pol-DNA interactions (Supplementary Figure S5D). The triphosphate tails of the incoming dTTP or dATP in all three complexes were coordinated to the B metal ion in a tridendate coordination geometry. Our previous structural studies with various RB69pol constructs have shown that the 5′ template overhang can adopt different conformations. Interestingly, for the three structures reported here, the 5′ template overhangs were completely superimposable, and the nucleotide residues at position n + 1 of the template strands were stabilized by stacking on top of the phenyl ring of F359.

### Structural basis for the kinetic behavior of L415 variants

**Figure 5.**
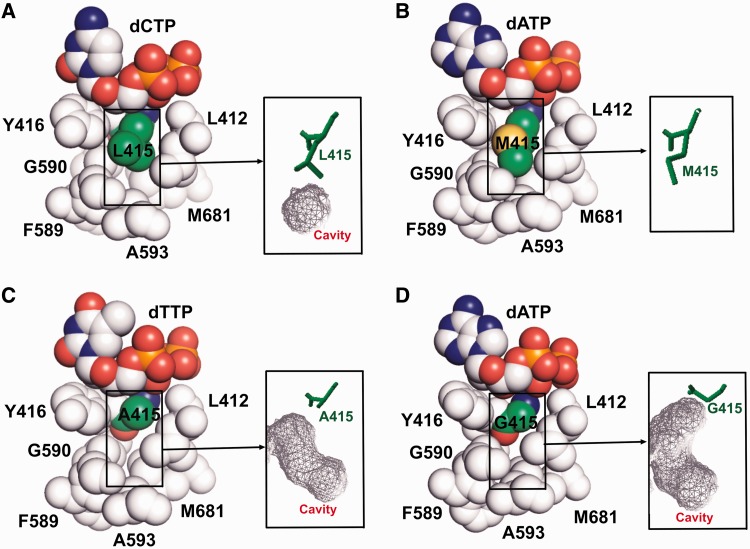
Space-filling model showing residues around (**A**) L415, (**B**) M415, (**C**) A415 and (**D**) G415. The size of the cavity adjacent to residue 415 is shown in gray mesh.

**Figure 6.**
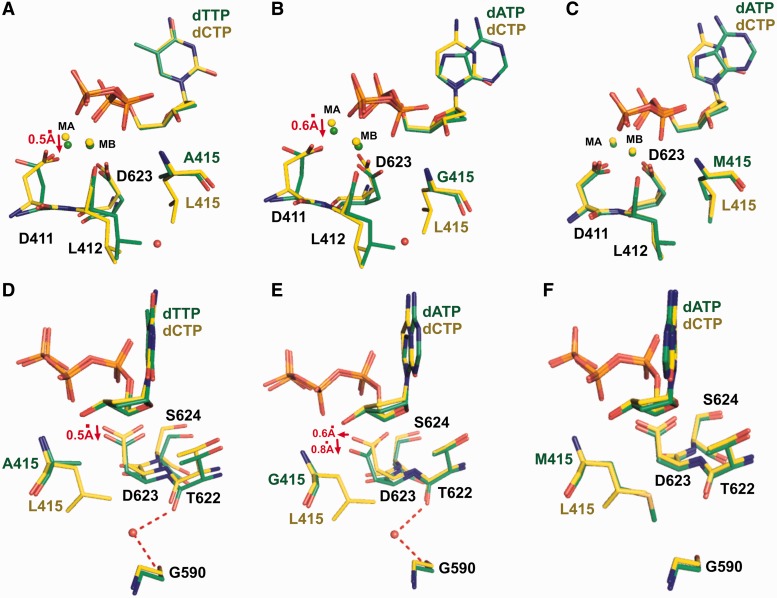
Superposition of the ternary structure of wt RB69pol with that of (**A**) L415A, (**B**) L415G, (**C**) L415M, (**D**) L415A, (**E**) L415G and (**F**) L415M variants in their respective ternary complexes. The wt RB69pol is shown in yellow sticks, and L415 variants are shown in green sticks. Panel (A)–(C) are related to panel (D)–(F) by a 90° rotation.

**Figure 7.**
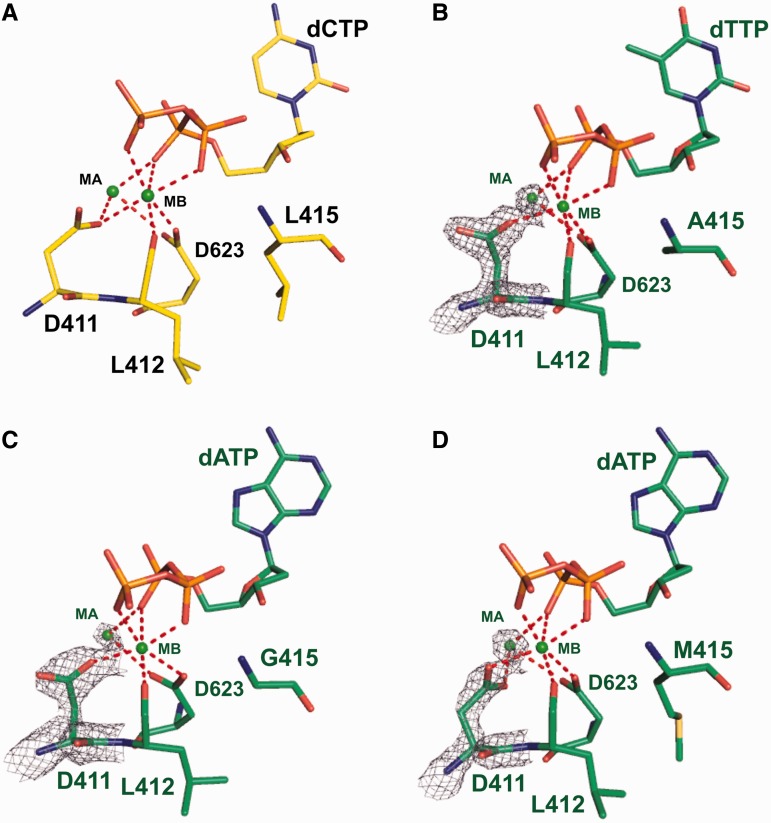
The nucleotide binding pocket of; (**A**) wt RB69pol; (**B**) L415A; (**C**) L415G and (**D**) L415M. The wt RB69pol is shown as yellow sticks, and L415 variants are shown as green sticks. Final 2F_o_-F_c_ electron density map around metal ion A and D411 of the L415 variants are contoured at 2.0 σ. Atoms within a 3.4 Å distance to metal ion A or B are linked with a red dashed line.

A space-filling representation of residues around L415 in the structure of wt RB69pol ternary complex shows that there is a small hydrophobic cavity at the tip of the L415 side chain (Figures 1 and 5A). Replacing L415 with either Ala or Gly greatly enlarges the size of this cavity (Figure 5C and D). Superposition of the L415A ternary complex with our previous reported dGTP/dC-containing wt ternary complex shows that; (i) the side chain of D411 adopts a rotamer conformation distinctively different from that of wt RB69pol (Figure 6A) and is no longer coordinated to metal ion A (Figure 7A and B); (ii) metal ion A shifts vertically by 0.5 Å away from the primer terminus (Figure 6D); (iii) the side chain of L412 tilts toward the cavity generated by L415 to Ala substitution (Figure 6A); (iv) one ordered water molecule appears in this cavity and is hydrogen-bonded to both the carbonyl oxygen of G590 and T622 (Figure 6D and; (v) the carboxyl group of D623 together with the main chains of T622, D623 and S624 shift vertically by 0.5 Å away from the incoming dNTP (Figure 6D). Similar differences were observed between structures of the L415G ternary complex and the wt RB69pol ternary complex (Figures 6B and E and 7C). In particular, the change of the metal ion A position in both the L415A and L415G ternary complexes are mediated by an alteration in peptide backbone (Figure 6A and B). The only discrepancy is that the backbones of T622 and S624 in the L415G ternary complex did not shift away from the position observed in wt RB69pol, instead the carboxyl group of D623 titled toward the cavity generated by the L415 to Gly substitution.

There is a common consensus that metal ion A plays a critical role in aligning the 3′ hydroxyl group (-OH) at the primer terminus and the α-phosphorus atom (Pα) of the incoming dNTP (37,38). The coordination bond length of metal ion A determines how close the 3′-OH can approach Pα (8). Therefore, the 0.5 Å downshifting of the metal ion A from its optimal position observed in the wt RB69pol structure would cause misalignment between the 3′-OH and Pα, possibly reducing the rate of phosphodiester bond formation. This could explain why the k_pol_ values for incorporating a correct dNMP by L415A and L415G mutants are 5- to 25-fold lower than the k_pol_ for wt RB69pol. The apparent dissociation constant (K_d,app_) reflects the balance between substrate binding and incorporation. Because the turnover rates decrease dramatically for the L415A and L415G mutants, the open and closed forms of the ternary complex likely reach equilibrium before chemistry (39,40). Thus, the K_d,app_ values for correct dNMP incorporation by the L415A and L415G mutants were 5- to 15-fold lower than the value determined with wt RB69pol. A similar scenario has been observed in the case of the RB69pol quadruple mutant, in which a 20-fold decrease in k_pol_ and 10-fold decrease in K_d,app_ compared with wt RB69pol correlates with the vertical shift of metal ion A observed in the structure of the qm ternary complex (15).

In contrast to the L415A and L415G mutants, the L415 to Met substitution did not disturb the adjacent residues. As shown in Figure 5B, the side chain of M415 is snuggly accommodated in the pocket surrounded by Y416, G590 and L412, thus fully occupying the cavity present in wt RB69pol. Superposition of the L415M ternary complex with the dGTP/dC-containing wt ternary complex shows that: (i) the side chain of D411 slightly tilts toward D623 (Figure 6C), as a consequence, each δ oxygen atom of D411 is coordinated to a metal ion (Figure 7D); (ii) the A metal ion is shifted slightly (<0.2 Å) away from the primer-terminus (Figure 6C); (iii) one of the δ carbon atoms of L415 overlays perfectly on top of the sulfur atom of M415 (Figure 6F); (iv) the carboxyl group of D623 deviates slightly from its orientation compared with wt RB69pol (Figure 6F). Interestingly, the rotameric position of D411 as observed in the wt structure is restored in the L415M mutant. This is in vivid contrast to the L415A and L415G mutants, where the Leu to Ala or, the Leu to Gly substitution disrupt the hydrophobic interaction between L412 and L415. This interaction anchors the D411 in the wt rotameric position. Overall, the structure of the L415M ternary complex is much closer to the structure of the wt RB69pol ternary complex than the structure of either the L415A or L415G ternary complexes. Accommodation of the M415 side chain in the hydrophobic cavity without disturbing the nearby residues could provide an energetic advantage to this mutant over wt RB69pol forming the conformation required for dNTP incorporation. A more rigid anchor of the metal ions would likely facilitate the alignment between the 3′-OH and Pα of the incoming dNTP. Together, these structural interpretations help explain the increased catalytic efficiency of the L415M mutant over wt RB69pol.

As the RB69pol qm enabled us to capture all mismatches in the nascent base pair binding pocket (NBP), we can now superimpose each dATP/dA, dCTP/dA and dTTP/dC-containing RB69pol qm structure with structures of the L415 variants. As shown in Supplementary Figure S6, all three mismatches can be modeled perfectly well into the NBP of L415A. In particular, the N3 of the templating dA is within HB distance of Cα of G568 (Supplementary Figure S6C). It is interesting that the three mismatches can be modeled into each of the three L415 variants without steric clashes. But it is puzzling why the L415A and L415G mutants are able to incorporate an incorrect dNMP ∼100 times more efficiently than the L415M mutant. We speculate that the main reason has to do with flexibility of the NBP. By replacing L415 with either Ala or Gly, the enlarged cavity provides extra space for nearby residues, such as L412 and Y416. The backbone amides of L412 and Y416 are hydrogen bonded to the β-phosphate and the 3′-OH of the incoming dNTP, respectively. Thus, the increased freedom of L412 and Y416 provides this extra flexibility for the incoming dNTP and allows it to adopt non-Watson–Crick geometry with the templating base. In contrast, the L415 to Met substitution would be expected to restrict the movement of nearby residues. As a consequence, the incoming dNTP would be rigidly anchored and could not accommodate the templating base to allow for non-Watson–Crick geometry.

Besides- L415, Y416 and D623 are highly conserved in the B family pols, as are the corresponding residues in Polδ (L612, Y613 and D764) (23); as well as in PolII (L423, Y424 and D547) (41) and in Phi29 pol (L253, Y254 and D458) (42). As shown in Supplementary Figure S7, the rotamer conformations of those residues are also conserved. For these reasons, we believe that the kinetic behavior and structural features observed for L415 variants of RB69pol should apply to other pols in the B family.

### Replacing L415 with Ala or Gly alters the conformational dynamics of the pol complexes

**Figure 8.**
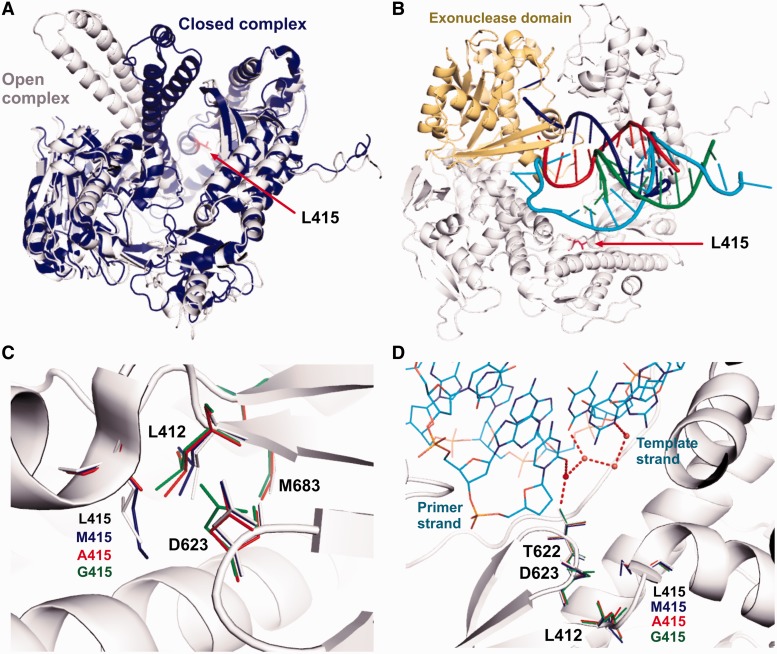
Structures of various RB69pol complexes. (**A**) Superposition of the structure of wt RB69pol closed ternary complex (PDB: 3NCI, shown in blue) with the structure of the binary complex in an editing mode (PDB: 1CLQ, shown in gray). The red arrow points to L415. (**B**) Shifting of P/T from pol to exo domain in wt RB69pol. The template strand in the pol domain is shown in cyan; the primer strand in the pol domain is shown in green. The template strand in the exo domain is shown in red; the primer strand in the exo domain is shown in blue. The exo domain is shown in yellow. (**C**) Superposition of the structures of all three L415 variants with that of wt RB69pol. Side chains in the structures of wt, L415M, L415A, L415G are shown in gray, blue, red and green, respectively. (**D**) A different view of C with emphasis on the interactions between T622 and the hydrogen bonding network located at the minor groove of the P/T junction.

It is believed that replicative DNA pols can bind DNA in at least two distinct conformations with the primer-terminus switching between the pol and exo domains. The relative partitioning of each conformation can be modulated by the identity of the primer-terminus. Steitz *et al.* reported a structure of a RB69pol binary complex in the editing mode, showing that the fingers domain rotated away from the palm by 60° (Figure 8A), and that the primer-terminus traveled 40 Å from the pol to exo domain (Figure 8B) (43,44). Our tC^o^-tC_nitro_ FRET assays show that replacement of L415 with Ala or Gly abolished the pol’s ability to differentiate fully complementary P/Ts from P/Ts with a mismatch at the P/T junction. Thus, it appears that the L415 to Ala or Gly substitutions alter the dynamics of pol to exo partitioning. Interestingly, L415 neither interacts with the P/T directly nor locates at the hinge region of the fingers domain (Figure 8A and B). The intriguing question then is why substitution of L415 with Ala or Gly affects pol-exo partitioning? It is unlikely that these mutants change the binding affinity of the pol-P/T complex, otherwise the FRET values for L415A-con-P/T or L415G-con-P/T binary complexes would be higher than those observed with the wt-con-P/T binary complexes. In addition, our previous kinetic studies with single, double and triple mutants of RB69pol showed that amino acid substitutions in the NBP of RB69pol does not affect the binding affinity to duplex DNA.

The structures of L415A and L415G mutants show that the L415 to Ala or Gly substitution greatly increases the size of the hydrophobic cavity that is surrounded by G590, F589, A593, M681 and L412 (Figures 1 and 5A). As a consequence, these residues gradually move in to partially occupy the vacated space, particularly the side chains of L412, D623 and M683 (Figure 8C). The degree of their tilting depends on the size of the cavity. For example, the L415G mutant has the largest cavity; thus, the corresponding side chains of L412 and D623 show the greatest tilt toward the cavity (Figure 8C). Adjacent to D623 is residue T622, which is hydrogen bonded to the O2 atom in pyrimidines or the N3 atom in purines via a water molecule when these bases are at the primer-terminus (Figure 8D). This water molecule is part of the rigid hydrogen bonding network located at the minor groove of the P/T junction. Our structures of RB69pol ternary complexes show that this HB network plays an important role in stabilizing the P/T in the pol domain by serving as an extension of the relevant side chains (11,17). Thus, the relaxation of residues around the cavity could extend to the P/T terminus through this HB network. In addition, a water molecule is observed in this hydrophobic cavity when L415 is replaced with Ala or Gly. Several theoretical studies have concluded that entry of a water molecule into a small hydrophobic cavity is energetically unfavorable (26,45–47). This implies that the trapped water observed in a hydrophobic cavity would have less freedom to rotate (48–50). In accord with these studies, this trapped water molecule is hydrogen bonded to both the carbonyl oxygen of T622 and G590. Interestingly, T622 and D623 are located at the loop region of a small β-hairpin, which is critical for alignment of the primer terminus and the incoming dNTP (Figure 8D). The water mediated HB between T622 and D623 would likely restrict the movement of this β-hairpin and could affect the stabilization of a P/T in the pol domain. This speculation is consistent with our tC^o^ and tC_nitro_ FRET assay results, as the FRET values for the L415A-con-P/T or the L415G-con-P/T binary complexes are much lower than that observed for the wt-con-P/T binary complex.

To summarize, we have shown that replacing L415 with Ala or Gly alters the conformational dynamics of pol complexes: (i) at the binary complex level, it affects the partitioning of P/Ts between pol and exo domains; (ii) at the ternary complex level, it reduces the maximum turn-over rate for incorporation of correct dNTPs and increases the catalytic efficiencies for incorporation of an incorrect dNTPs. Our structural data show that the L415 to Ala or Gly substitutions have at least three important effects: (i) they increase the size of the cavity located at the tip of the L415 side chain; (ii) they shift the metal ion A position; and (iii) they change the rotamer conformation of critical carboxyl groups. In addition to the kinetic and structural studies, we developed a novel tC^o^-tC_nitro_ FRET assay, which has the potential to be used with pols in other families, to monitor the partitioning of the primer-terminus between the pol and exo domains. The assay is relatively simple compared with the previously reported near ultraviolet circular dichroism assay (51), which requires unusually long scanning times (≈24 h) to detect occupancy of the pol or exo domains based on small differences in the CD signals.

## SUPPLEMENTARY DATA

Supplementary Data are available at NAR Online.

## FUNDING

Funding for open access charge: National Institutes of Health [RO1-GM063276-09 to W.H.K.] and National Institutes of Health [R01-GM097348 to E.M.D.L.C.].

*Conflict of interest statement*. None declared.

## Supplementary Material

Supplementary Data
