# Supplementary material for: Alteration in the cavity size adjacent to the active site of RB69 DNA polymerase changes its conformational dynamics
Source: Nucleic Acids Res. 2013 Aug 5;41(19):9077–89. doi: 10.1093/nar/gkt674 (PMC3799440; doi:10.1093/nar/gkt674)
Supplement: Supplementary Data [file supp_41_19_9077__index.html]

Alteration in the cavity size adjacent to the active site of RB69 DNA polymerase changes its conformational dynamics — Alteration in the cavity size adjacent to the active site of RB69 DNA polymerase changes its conformational dynamics — Supplementary Data 

# Alteration in the cavity size adjacent to the active site of RB69 DNA polymerase changes its conformational dynamics

## 

files

**Files in this Data Supplement:**

- Supplementary Data - pdf file
