# Supplementary material for: Alteration in the cavity size adjacent to the active site of RB69 DNA polymerase changes its conformational dynamics
Source: Nucleic Acids Res. 2013 Aug 5;41(19):9077–89. doi: 10.1093/nar/gkt674 (PMC3799440; doi:10.1093/nar/gkt674)
Supplement: Supplementary Data [file supp_gkt674_nar-01544-h-2013-File010.pdf]

Alteration In The Cavity Size Adjacent To The Active Site of RB69 DNA Polymerase Changes Its Conformational Dynamics

Shuangluo Xia, Marcus Wood, Michael J. Bradley, Enrique M. De La Cruz, & William H. Konigsberg*

SUPPLEMENTARY MATERIALS

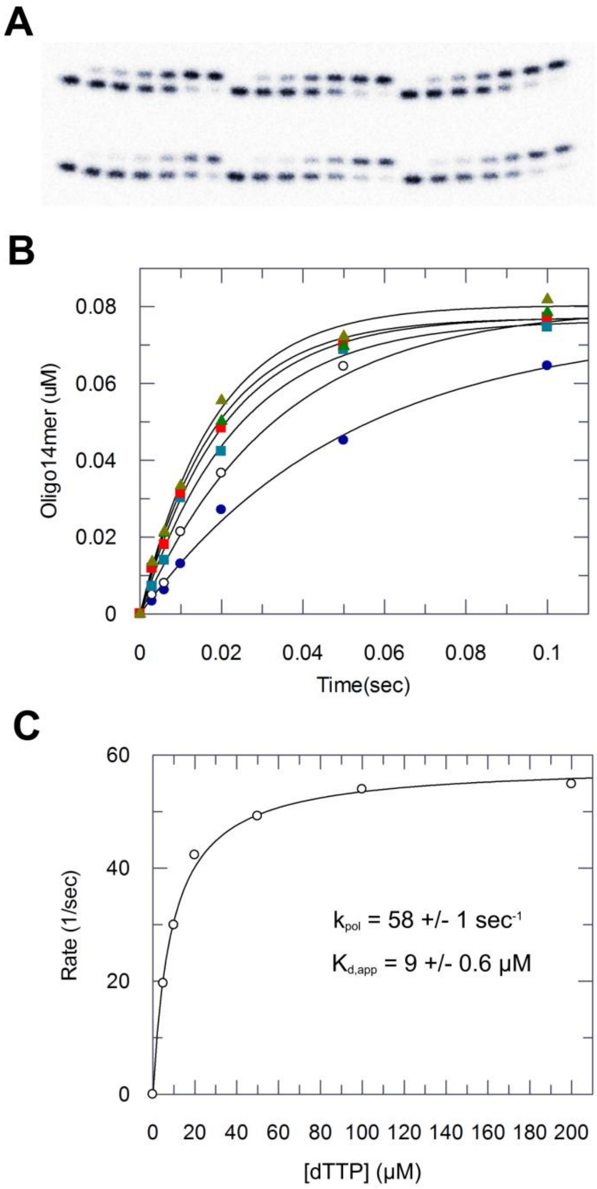

Figure S1. Raw data and plots for the insertion of dTMP opposite dA by the L415G mutant. (A) A denaturing PAGE gel showing the time course of dTMP incorporation with different [dTTP]: bottom lane (5μM, 10μM, 20μM from left to right) and top lane (50μM, 100μM, 200μM from left to right). (B) Progress curves at various [dTTP], 5, 10, 20, 50, 100, 200μM (from bottom to top), fit to a single-exponential equation. (C) Plot of k_{obs} vs [dTTP] fit to a hyperbolic equation to yield k_{pol} and $K_{d,app}$.

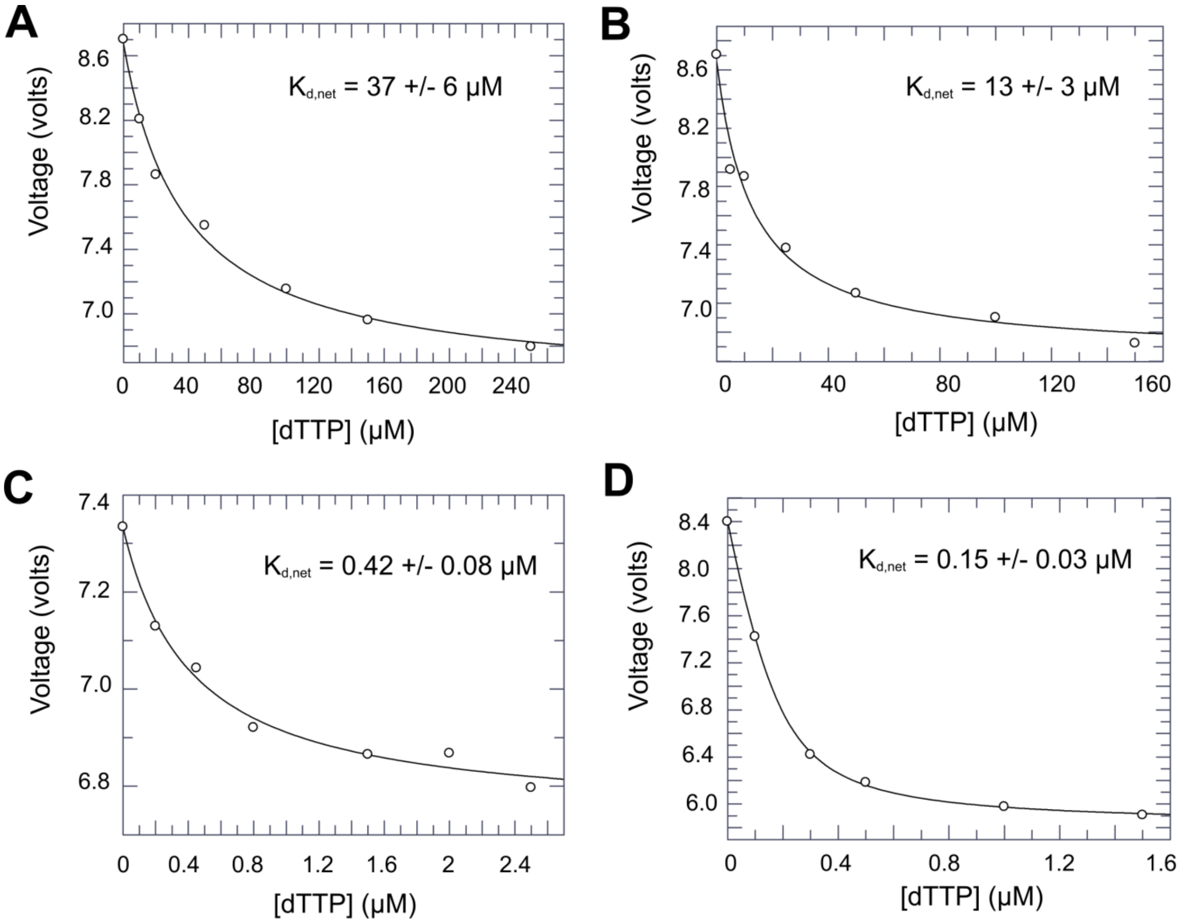

Figure S2. Plots of changes in amplitude obtained by stopped-flow fluorescence for dTTP opposite 2AP with; (A) wt RB69pol; (B) L415M; (C) L415G and L415A variants. Representative time courses of stopped-flow fluorescence for dTTP opposite 2AP with L415G mutant is shown in Figure S3.

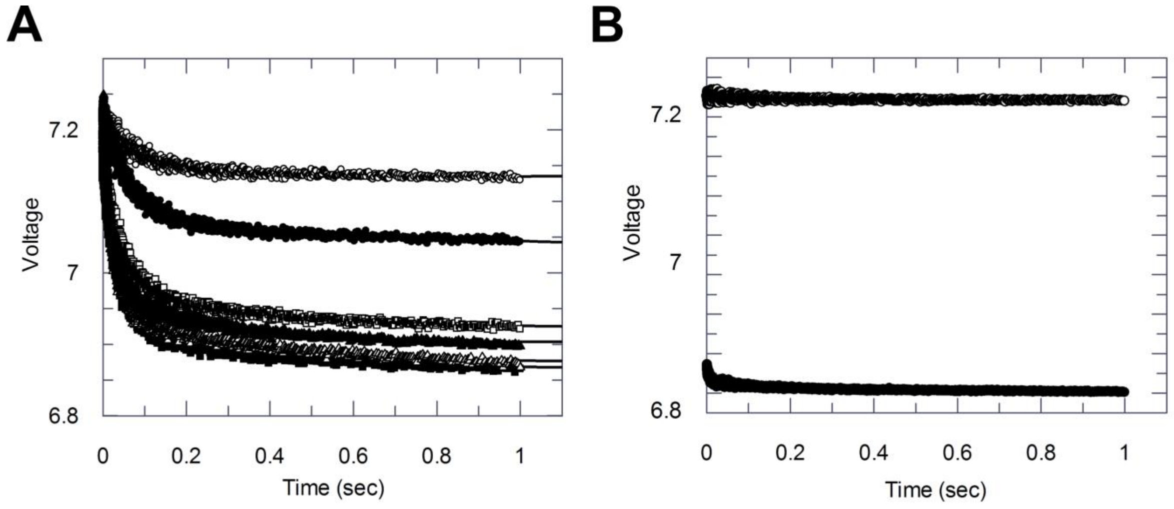

Figure S3. Stopped-flow fluorescence with dTTP opposite 2AP with L415G mutant. (A) Time courses of fluorescence quenching with different [dTTP], 0.2, 0.45, 0.8, 1.5, 2, 2.5μM (from top bottom), fit to a double-exponential equation. (B) Time courses of fluorescence quenching in the absence of dTTP (top) and in the presence of 100μM dTTP (bottom).

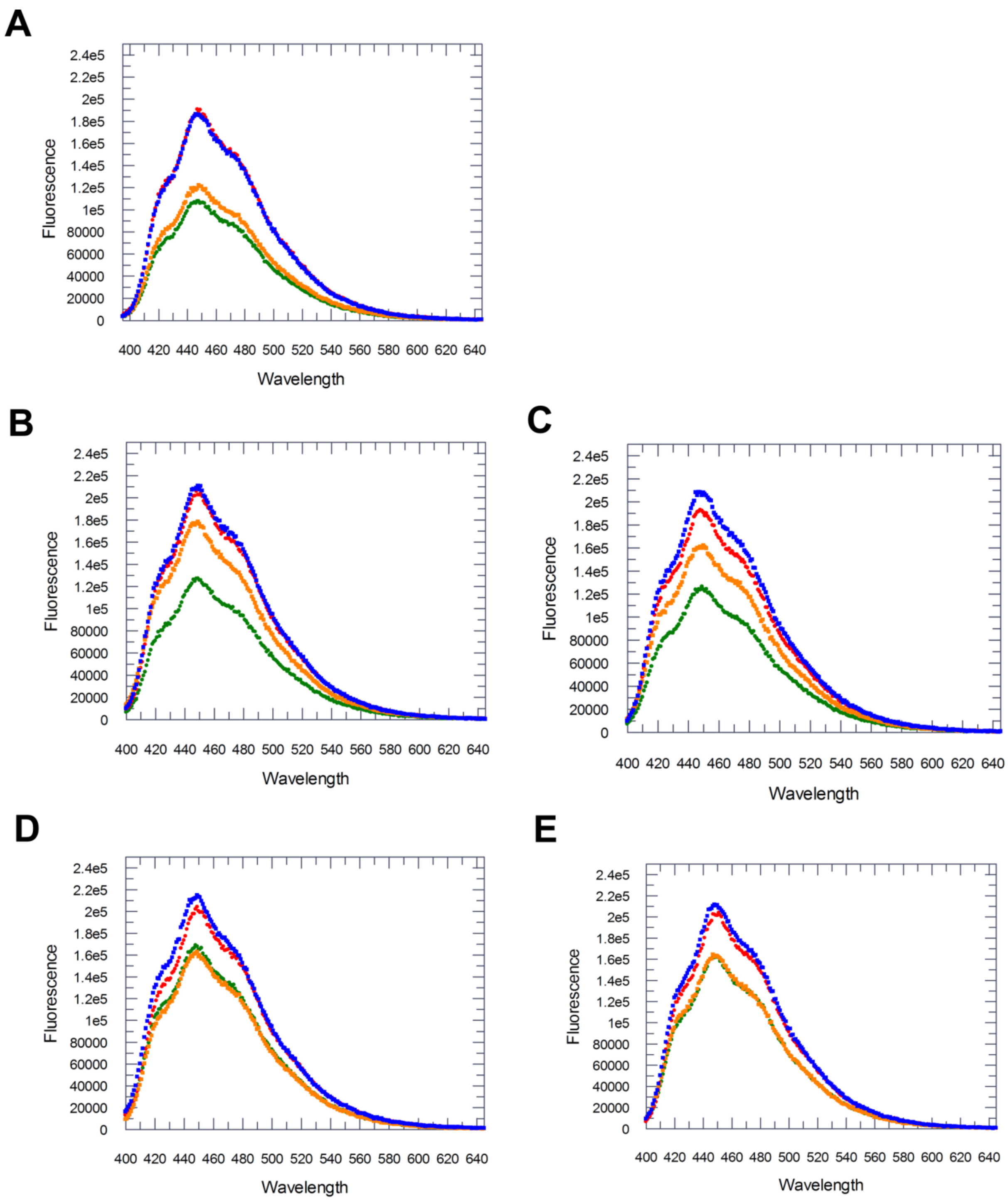

Figure S4. Fluorescence spectra of various tC^{o}-containing P/Ts in the absence and presence of pol: (A) P/Ts alone; (B) P/Ts with wt RB69pol; (C) P/Ts with L415M mutant; (D) P/Ts with L415A mutant and (E) P/Ts with L415G mutant. Sequences of P/Ts used are shown in Figure 2C. Red dotted line is the spectrum with Con-P/T; green dotted line is the spectrum with Con-P/T-tC_{nitro}; blue dotted line is the spectrum with Mis-P/T; and orange dotted line is the spectrum with Mis-P/T-tC_{nitro}.

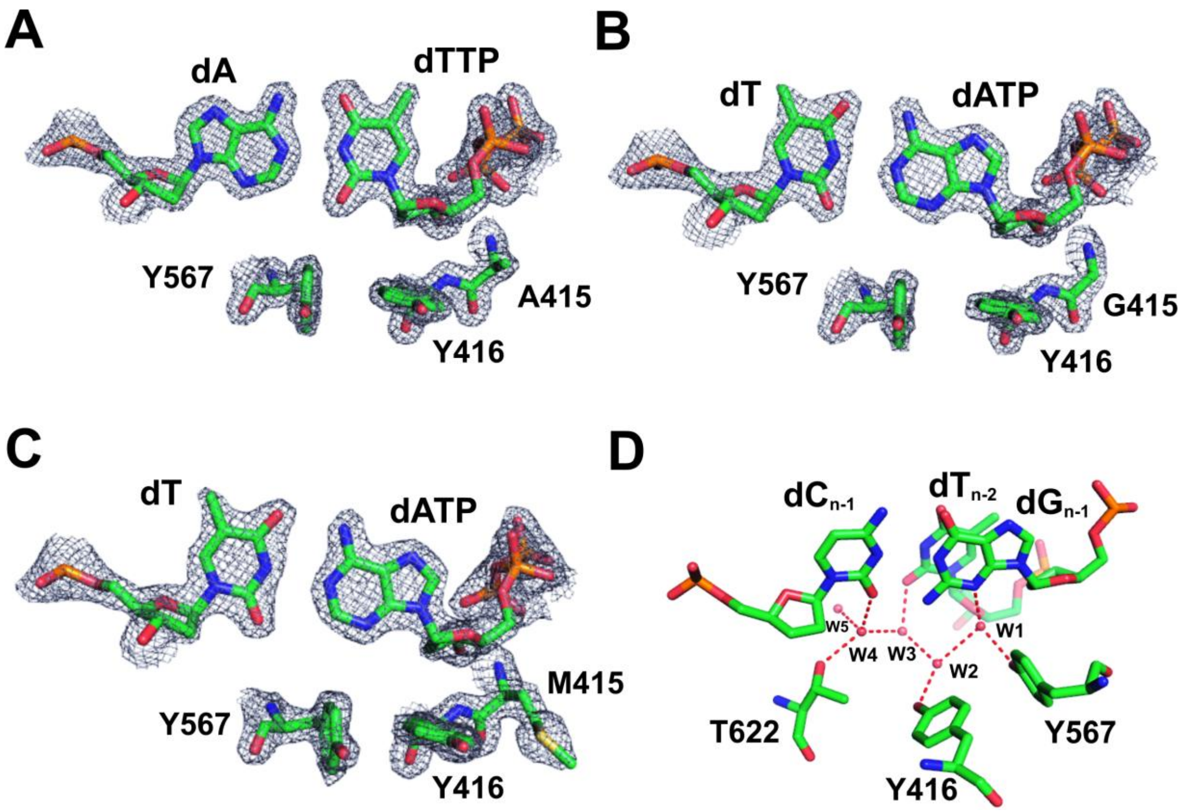

Figure S5. Structures of NBP of L415 variants. (A) Final $2F_o$-F_c electron density map at 1.80 Å resolution for the dTTP/dA-containing L415A complex contoured at 1.8 σ. (B) Final $2F_o$-F_c electron density map at 2.04 Å resolution for the dATP/dT-containing L415G complex contoured at 1.8 σ. (C) Final $2F_o$-F_c electron density map at 2.02 Å resolution for the dATP/dT-containing L415M complex contoured at 1.8 σ. (D) The hydrogen-bonding network at the minor groove of the P/T junction of L415A complex.

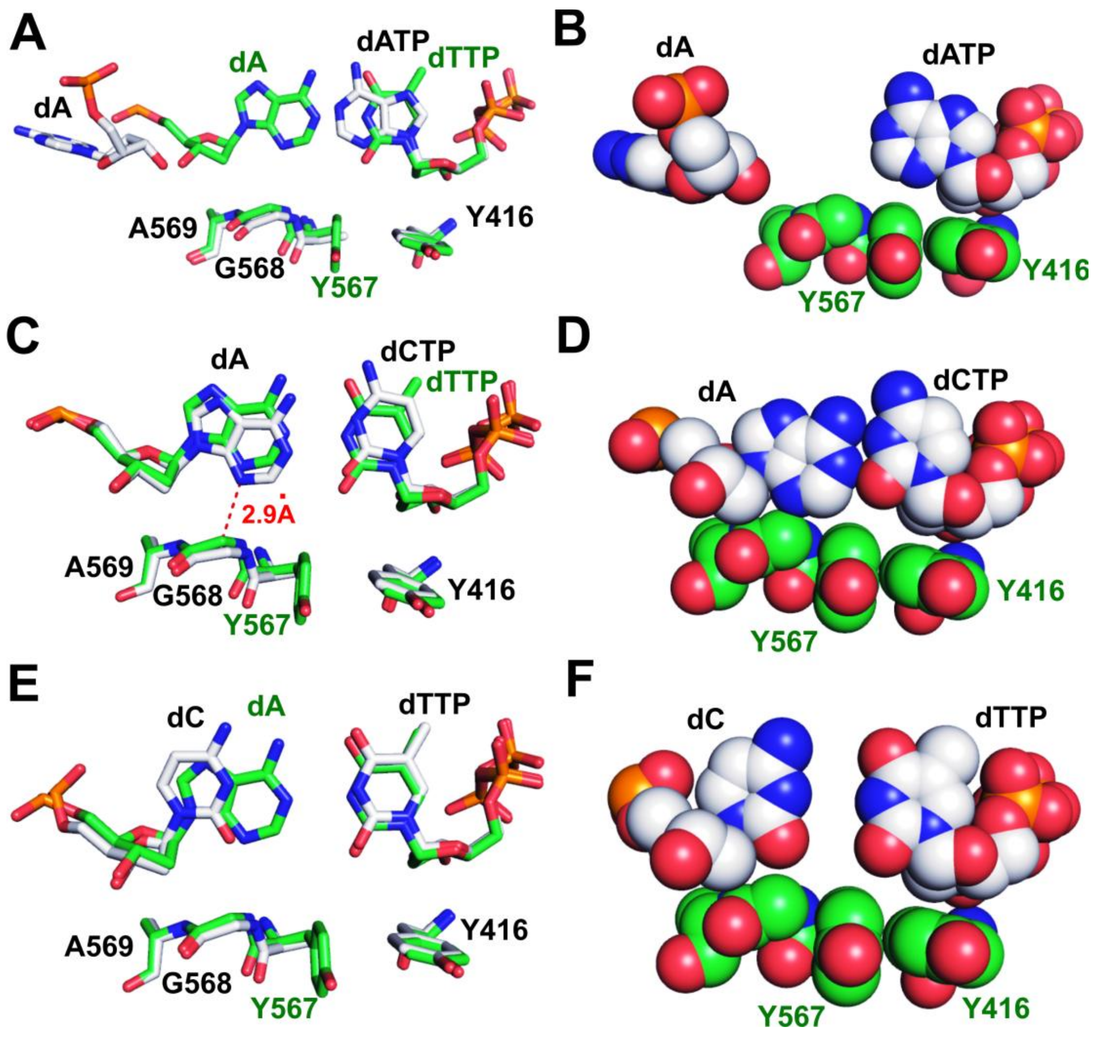

Figure S6. Superposition of mismatches-containing structures of the qm RB69pol onto the dTTP/dA-containing L415A. The structure with a mismatch is shown in gray, and the L415A structure is shown in green. (A) dATP/dA; (C) dCTP/dA and (E) dTTP/dC. (B), (D) and (F) are space-filling modes of (A), (C) and (E) respectively.

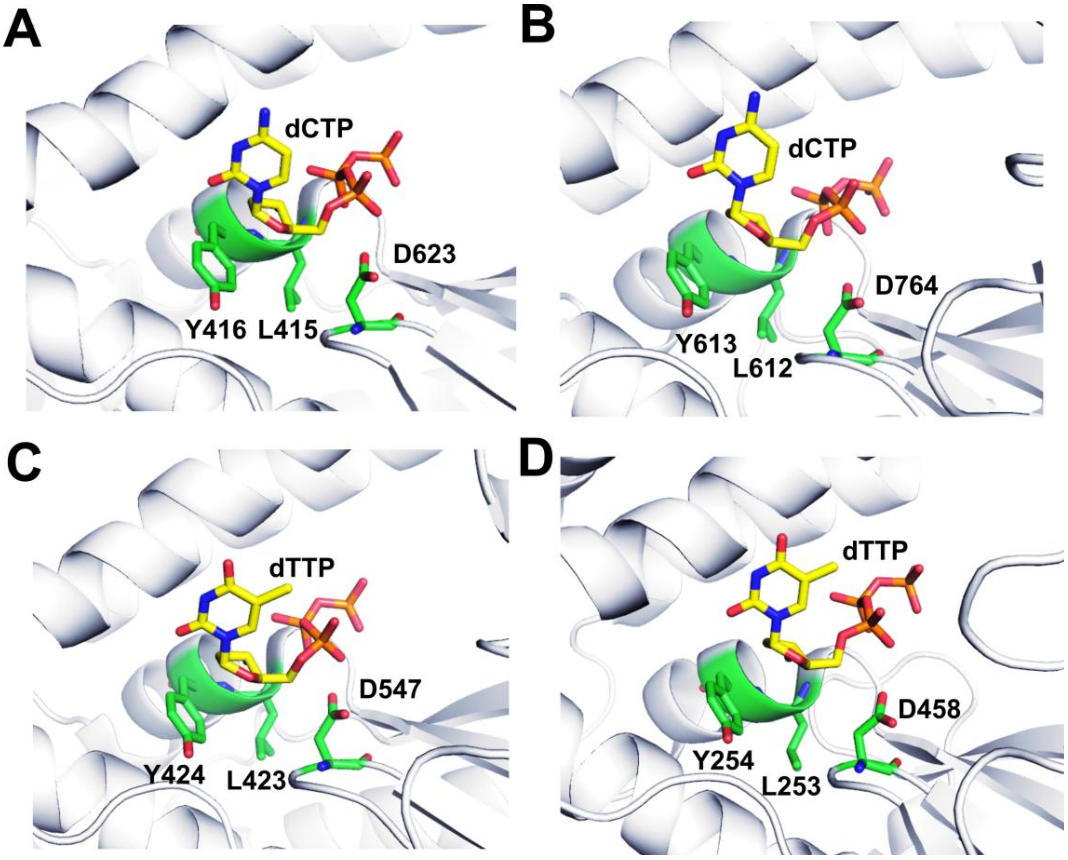

Figure S7. Conserved NBP residues in (A) RB69pol, (B) pol δ, (C) polII, and (D) Phi29 DNA pol.
